# Ultrasound-guided percutaneous cholecystostomy for acute cholecystitis: a systematic review and meta-analysis

**DOI:** 10.1007/s40477-026-01119-x

**Published:** 2026-02-10

**Authors:** Andrea Boccatonda, Alice Brighenti, Marco Musmeci, Nicola Venturoli, Livia Masi, Daniela Agostinelli, Sofia Maria Bakken, Susanna Vicari, Cosima Schiavone, Carla Serra

**Affiliations:** 1 Diagnostic and Therapeutic Interventional Ultrasound Unit, IRCCS Azienda Ospedaliero-Universitaria di Bologna, Policlinico Sant’Orsola-Malpighi, Via Massarenti N 9, 40138 Bologna, Italy; 2 Department of Medicine and Science of Aging, Università degli Studi Gabriele d’Annunzio Chieti Pescara Scuola di Medicina e Scienze della Salute, Chieti, Italy

**Keywords:** Gallbladder, Drain, Cholecystitis, Cholecystostomy, Ultrasound

## Abstract

**Background:**

Ultrasound-guided percutaneous cholecystostomy (US-PC) is widely used in high-risk surgical patients with acute cholecystitis. Still, success and safety rates specific to US guidance are not always distinguished within mixed 'image-guided' series.

**Objective:**

To estimate technical success, clinical success, and major complication rates of US-PC. Methods. Primary studies explicitly reporting US-PC or with separable data were included. Primary outcomes: technical success, clinical success (as defined by the study), and major adverse events (AEs). Single-arm meta-analysis using random-effects (logit, continuity correction); heterogeneity by I2 and prediction interval. Results. Four eligible single-arm studies for technical success (*N* = 466). Pooled technical success 99.3% (95%CI 97.8–99.8; I2 = 0%). Three studies (*N* = 223) reported clinical success: pooled 97.6% (95%CI 83.4–99.7; I^2^≈64% due to varying definitions). Two studies (*N* = 293) reported major AEs: pooled 1.7% (95%CI 0.7–4.1; I^2^ = 0%). Thirty-day mortality is available from one large study (6.2%). Conclusions. In high-risk patients, US-PC demonstrates very high technical success and low major complication rates; clinical success is high but variably defined across studies. Further direct comparisons and standardized definitions are warranted.

**Supplementary Information:**

The online version contains supplementary material available at 10.1007/s40477-026-01119-x.

## Introduction

**Fig. 1:**
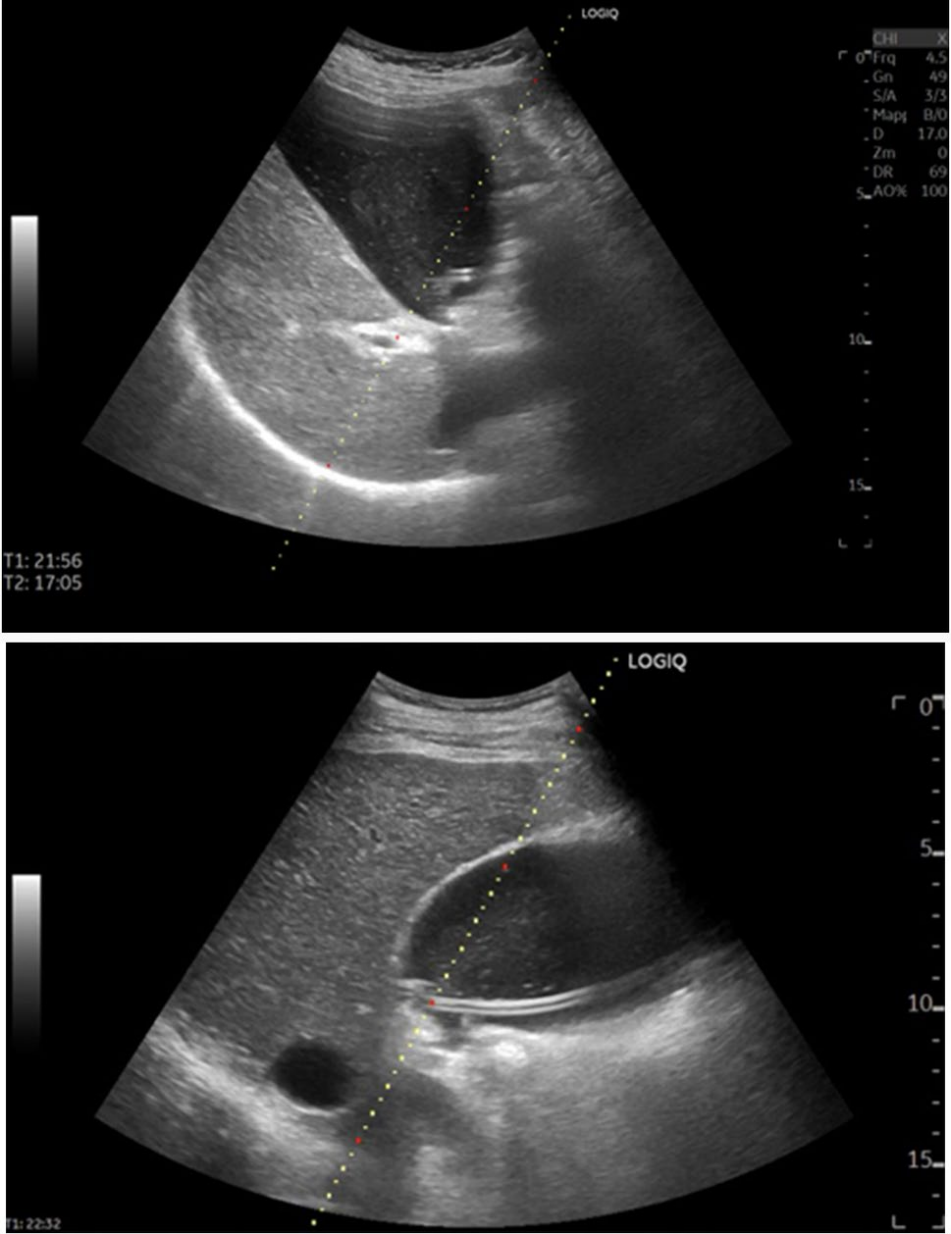
Screen right-upper-quadrant ultrasound with grayscale B-mode. An 8-Fr percutaneous pigtail drain is visible within the gallbladder (echogenic tubular structure with intraluminal course), consistent with transhepatic cholecystostomy; the pigtail tip is seen curled in the lumen. Coexisting gallstones produce echogenic foci with posterior acoustic shadowing

Acute cholecystitis is a common emergency with rising incidence in aging and multimorbid populations [1]. While early laparoscopic cholecystectomy is the standard of care for most patients, a substantial subset—particularly those with severe physiologic derangement, advanced frailty, or prohibitive anesthetic risk—are not immediate surgical candidates [1, 2]. For these patients, decompression of the inflamed gallbladder with percutaneous cholecystostomy (PC) can provide rapid source control, stabilize sepsis, and serve either as a bridge to elective cholecystectomy or as definitive therapy [1–4]. Among image-guided approaches, ultrasound-guided PC (US‑PC) offers several practical advantages: portability, avoidance of ionizing radiation and iodinated contrast, and the possibility of true bedside procedures in the intensive care unit [3, 5, 6]. Real-time visualization of the needle path and gallbladder dynamics can facilitate safe transhepatic track creation and catheter placement (Fig. 1).

Conversely, computed tomography–guided PC (CT‑PC) may be preferred when acoustic windows are poor, anatomy is complex, or concomitant intra‑abdominal pathology requires cross‑sectional delineation [7]. Despite widespread use, published series often pool image guidance modalities, limiting modality‑specific estimates of efficacy and safety. This systematic review focuses specifically on US‑PC, quantifying technical and clinical success and major adverse events across contemporary cohorts. We also provide an exploratory, hypothesis‑generating juxtaposition with CT‑PC using clearly separable single‑center cohorts, acknowledging the inherent limitations of indirect comparisons and potential confounding by indication. This systematic review aims not only to quantify outcomes specific to US-PC but also to highlight persistent areas of uncertainty that warrant further investigation. Our goal is to support evidence‑informed decision‑making for high‑risk patients and to highlight priority areas for standardization and comparative research.

Methods

### Reporting guideline

This review adheres to PRISMA 2020. A protocol was drafted a priori (scope, eligibility, outcomes, analyses) and served as guidance for the conduct of the review.

### Eligibility criteria

Population: adults (≥ 18 years) with acute cholecystitis (calculous or acalculous), any care setting; Tokyo severity grade recorded when available. Intervention: ultrasound-guided percutaneous cholecystostomy (US-PC) performed with real-time ultrasound guidance (any access route, catheter size, bedside or suite). Comparator: not required for primary single-arm outcomes; when present, any active comparator (medical therapy, early cholecystectomy, other drainage) recorded but not synthesized here.

Outcomes (primary): technical success; clinical success (per study definition). Outcomes (secondary): major adverse events (AEs) as per study/guideline definitions, 30-day or in-hospital mortality, specific AEs (bleeding, bile leak, dislodgement), length of stay, readmission, recurrence, interval cholecystectomy. Study designs: randomized or nonrandomized comparative studies and single-arm cohorts/series (≥ 10 participants). Setting/Time/Languages: no restriction on setting, year, or language; translations performed if needed.

### Information sources and search strategy

Databases: MEDLINE (PubMed), Embase, Scopus, Web of Science, and Cochrane. Grey literature: ClinicalTrials.gov and WHO ICTRP; major conference abstracts (DDW, UEGW, ACG) for the last 5–7 years. The search covered the inception of each database to the last update. Key terms combined subject headings and text words for cholecystitis and US-PC, with filters for study designs. Complete search strings are provided in the Supplemental material.

### Selection process

Two reviewers independently screened titles/abstracts and full texts against eligibility criteria, using standardized forms. Disagreements were resolved by discussion or a third reviewer. Reasons for exclusion at full text were recorded. Deduplication was performed before screening. The PRISMA flow diagram reports numbers at each stage.

### Data collection process

Two reviewers independently extracted data using a piloted template (study characteristics, patient demographics, severity, technique including access route and device, outcomes, follow-up, funding/conflicts). When data were missing or unclear, study authors were contacted where feasible. If multiple reports described the same cohort, the most complete/updated dataset was used and overlapping data were avoided.

### Data items

We predefined all data items, including definitions for technical success (successful catheter placement with bile aspiration) and clinical success (study-defined resolution by 72–96 h or predefined biochemical response). Major AEs followed study/guideline definitions (e.g., sepsis, hemorrhage requiring transfusion/intervention, abscess/biloma, procedure-related death). We extracted denominators for each outcome (e.g., per-procedure for technical success vs. per-patient for clinical success) and the exact time windows (30-day vs. in-hospital).

### Risk of bias assessment

Risk of bias for nonrandomized studies was planned with ROBINS-I across seven domains (confounding, selection, classification of interventions, deviations, missing data, outcome measurement, reporting). Given that the included evidence was predominantly single-arm cohorts, applicability and reporting biases were qualitatively appraised. Risk-of-bias judgments were intended to inform sensitivity analyses and GRADE certainty ratings.

### Effect measures

For dichotomous single-arm outcomes (technical success, clinical success, major AEs, mortality), the effect measure was the proportion with 95% confidence intervals. For comparative outcomes (not synthesized here), risk ratios (RR) or odds ratios (OR) were prespecified.

### Synthesis methods

We performed single-arm meta-analyses using a random-effects model (DerSimonian–Laird) on logit-transformed proportions with continuity correction (+ 0.5 to events and non-events). We reported pooled estimates with 95% confidence intervals and 95% prediction intervals. Statistical heterogeneity was assessed with Q and I2 statistics. Where definitions were heterogeneous (e.g., clinical success), findings were interpreted with caution.

### Reporting bias and small-study effects

Given the small number of included studies per outcome (*k* < 10), formal assessment of small-study effects or publication bias (e.g., funnel plots/Egger) was not performed or would be underpowered. We considered selective reporting qualitatively during data extraction.

### Certainty assessment (GRADE)

Certainty of evidence for each critical outcome was judged using GRADE, starting from low certainty for nonrandomized evidence and rating down for risk of bias, inconsistency, indirectness, and imprecision as applicable. Summary of Findings (SoF) tables were produced, with explanatory footnotes documenting judgments.

## Results

### Study selection and characteristics

**Fig. 2:**
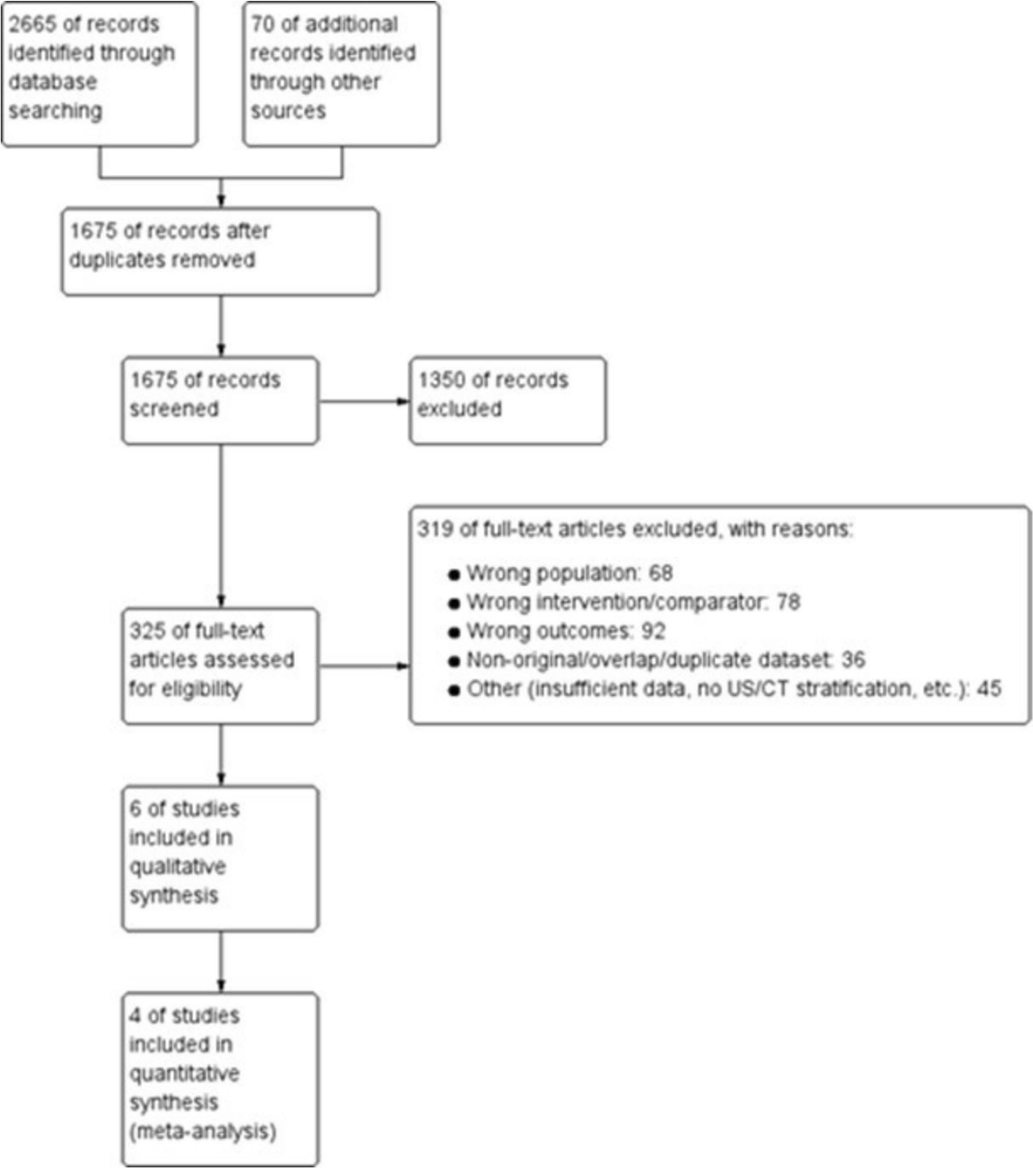
Study flow diagram

The search yielded 2,665 records from databases and 70 from other sources (reference lists, conference abstracts, citation tracking). After deduplication, 1,675 records remained and were screened by title/abstract; 1350 were excluded as irrelevant. We assessed 325 full‐text articles for eligibility. Of these, 319 were excluded for the following reasons: wrong population (*n* = 68), wrong intervention/comparator (*n* = 78), wrong outcomes (*n* = 92), non-original/overlap/duplicate dataset (*n* = 36), and other reasons (insufficient data, no US/CT stratification, etc.; *n* = 45). In total, 6 studies were included in the qualitative synthesis [8–13], and 4 studies contributed data to the quantitative meta-analysis [8–11] (Fig. 2).

Four primary cohorts explicitly reporting US‑PC were included for quantitative synthesis [8–11] (Table 1). All studies enrolled adults with acute cholecystitis in high‑risk settings [8–11]; three were single‑center series, and one was a large single‑center cohort [9]. US‑PC was predominantly performed via a transhepatic approach with small‑caliber pigtail catheters (8–10 Fr). One cohort was conducted entirely at the ICU bedside [10], whereas others were performed in interventional radiology suites [8, 9, 11]. Clinical success definitions varied: symptom‑based resolution within ~ 72 h in two studies [8, 10] versus a biochemical response (≥ 25% decline in CRP/direct bilirubin at 72 h) in one study [11].

**Table 1:** Characteristics and outcomes of ultrasound-guided transhepatic percutaneous cholecystostomy across four cohort studies

Author (year)	Country	*N*	Access/technique	Catheter (F)	Technical success	Major findings
Bakkaloglu (2006) [8]	Turkey	27	US, transhepatic, Seldinger	10	27/27 (100%)	Clinical success 27/27; 3 dislodgements, 1 bleed; 5 interval cholecystectomy
Kesim (2023) [11]	Turkey	145	US, transhepatic	8	145/145 (100%)	Clinical success 145/145 (biochemical); 52% definitive, 48% interval surgery
Gandhi (2024) [10]	India	51	US, transhepatic (ICU bedside)	NA	51/51 (100%)	Clinical success 47/51 (92%); no major AEs; 1 bile leak
Dewhurst (2012) [9]	USA	243	US, transhepatic 95%	8	242/243 (99.6%)	Major AEs 4/242 (1.7%); 30-d mortality 15/242 (6.2%)

Columns report country, sample size (*N*), access/technique, catheter caliber (French), technical success, and key clinical findings (as defined by the original authors), including complications and 30-day outcomes where available

*US* ultrasound; *ICU* intensive care unit; *AEs* adverse events; *NA* not available

### Risk of bias in studies

**Fig. 3:**
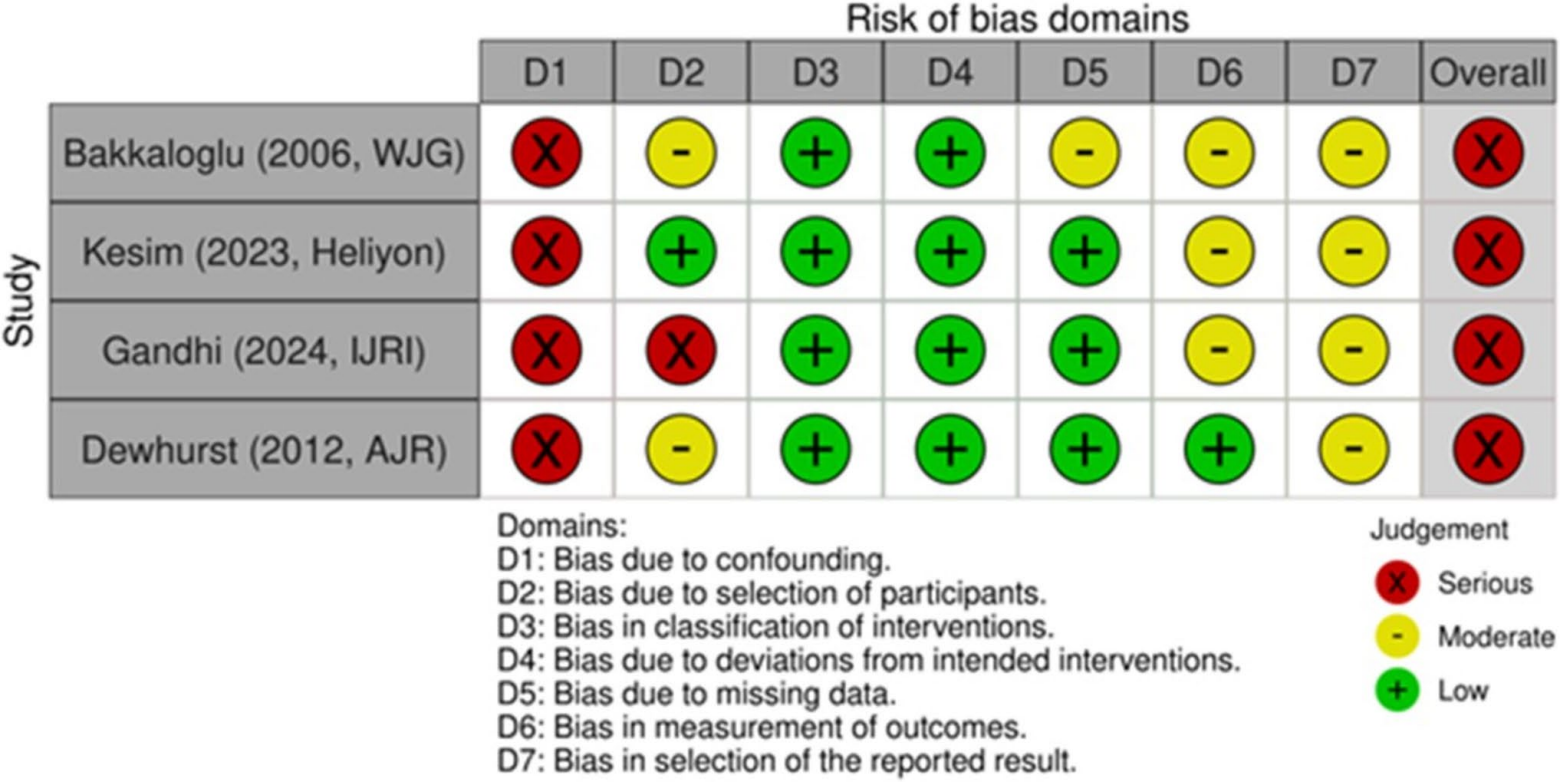
ROBINS‑I domain summary (RobVis)

**Fig. 4:**
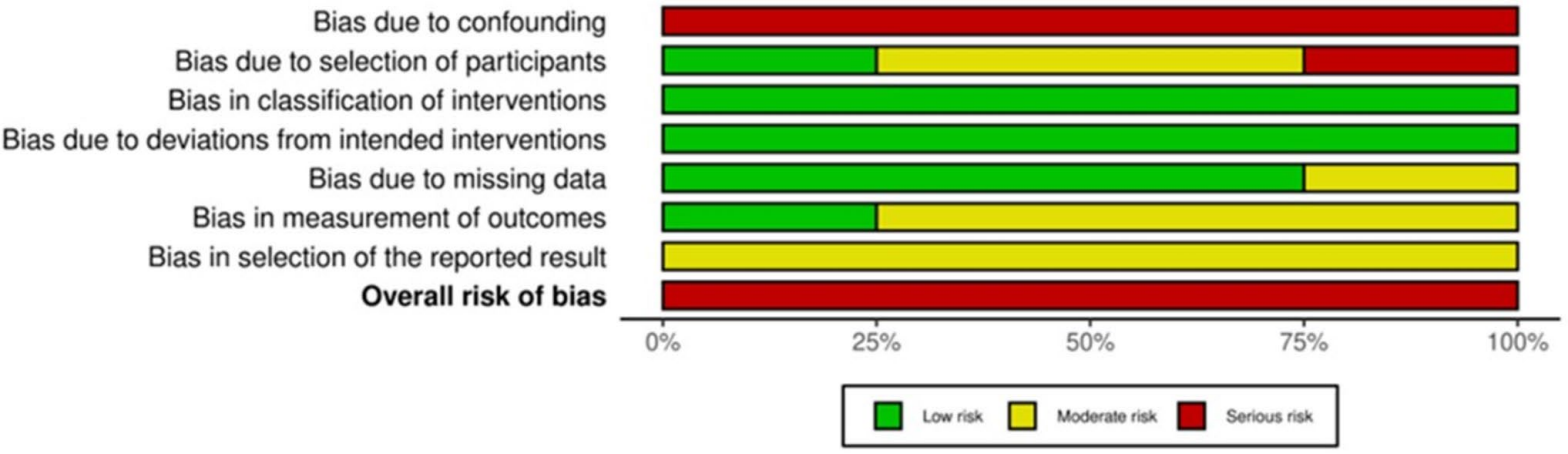
ROBINS‑I traffic‑light plot by study (robvis)

We assessed risk of bias using ROBINS-I for non-randomized studies across seven domains (Figs. 3 and 4). Two reviewers conducted independent assessments with disagreements resolved by consensus. Study-level and domain-level summaries are shown in the figures. All included studies [8–11] were rated as having an overall “serious” risk of bias, driven primarily by confounding (D1). The observational design, lack of randomization, and limited adjustment for key covariates (e.g., patient characteristics, disease severity, lesion size/location, operator experience) mean that unmeasured differences likely influenced outcomes. The high risk from confounding—and, to a lesser extent, from outcome measurement—lowers confidence in the pooled estimates of accuracy/effect. Findings should be interpreted cautiously.

### Results of syntheses

#### Technical success

**Fig. 5:**
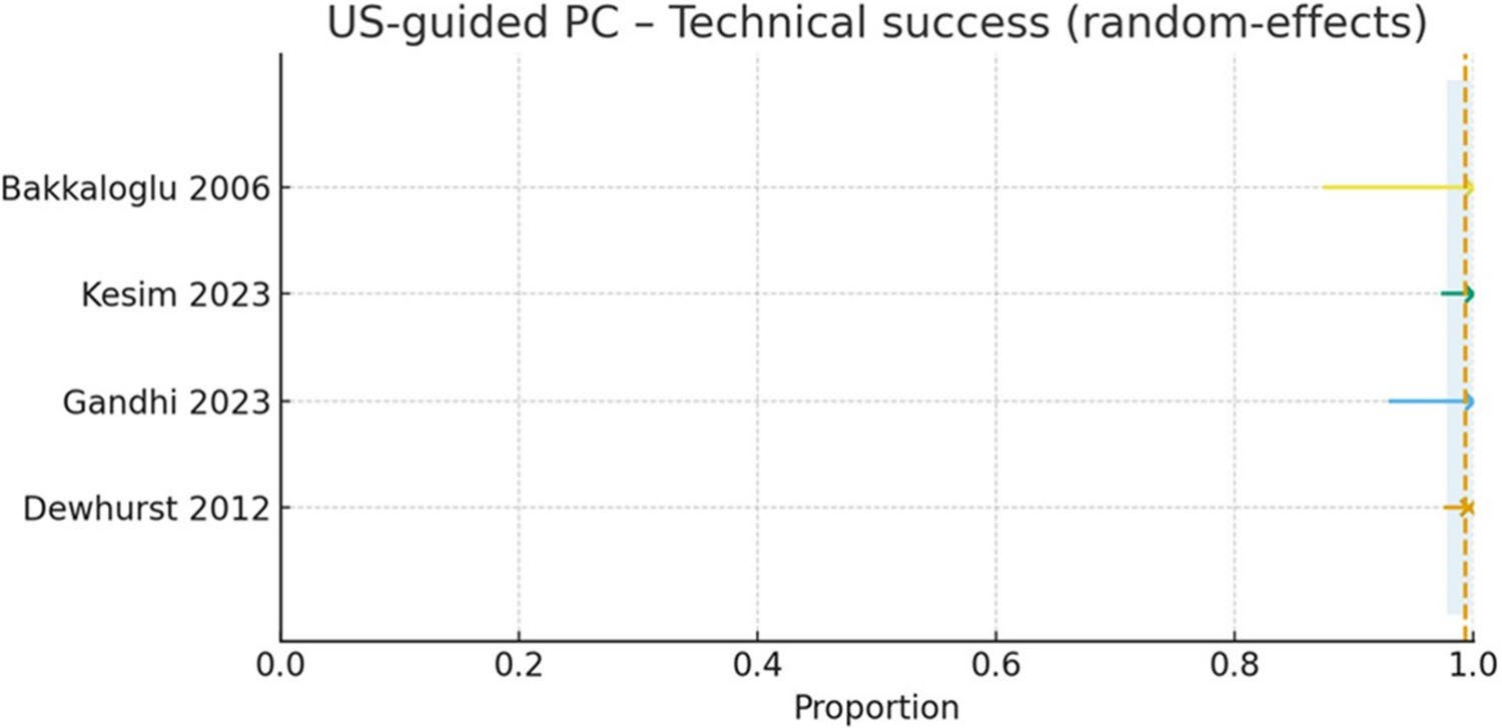
Study-level technical success of ultrasound-guided percutaneous cholecystostomy (US-guided PC) with a random-effects model. Each horizontal line shows the study estimate (point) with its 95% CI (Bakkaloglu 2006; Kesim 2023; Gandhi 2023; Dewhurst 2012). The vertical dashed line denotes the pooled proportion, and the shaded band its 95% CI. Across cohorts, technical success was consistently very high (≈95–100%)

Across 4 studies (*n* = 466 procedures), technical success was achieved in 465/466 cases (raw proportion 99.6%) [8–11] (Fig. 5). The random‑effects meta‑analysis (logit transformation with continuity correction) yielded a pooled technical success of 99.3% (95% CI 97.8–99.8; I2 = 0%), with a narrow prediction interval reflecting the consistency of results across centers. Individually, Bakkaloglu 2006 [8], Kesim 2023 [11], and Gandhi 2024 [10] each reported 100% technical success, while Dewhurst 2012 reported 242/243 (99.6%) [9].

#### Clinical success

**Fig. 6:**
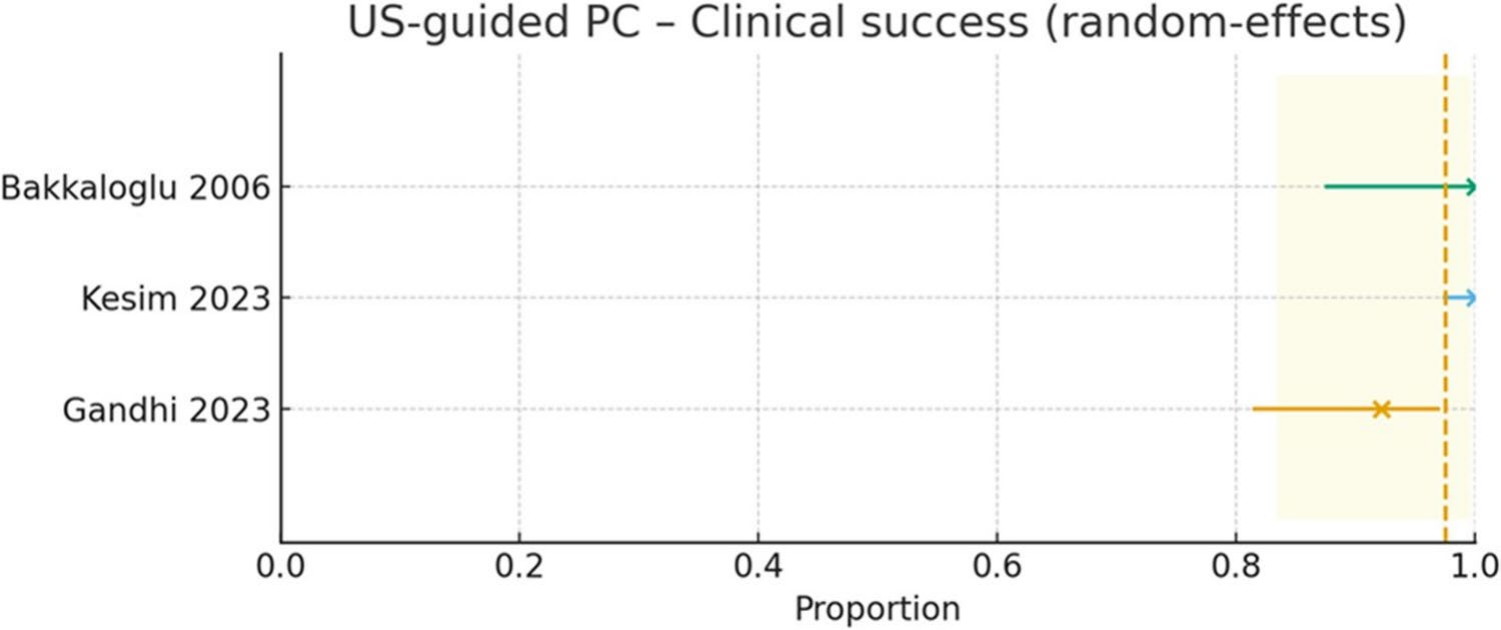
Study-level clinical success of ultrasound-guided percutaneous cholecystostomy (US-guided PC) with a random-effects model. Each horizontal line shows the study estimate (point) with its 95% CI (Bakkaloglu 2006; Kesim 2023; Gandhi 2023). The vertical dashed line indicates the pooled proportion, and the shaded band its 95% CI. Overall clinical success is high (≈90–100%), acknowledging that definitions of “clinical success” differed across cohorts

Three studies (*n* = 223) reported clinical success (Fig. 6): 27/27 (Bakkaloglu 2006) [8], 145/145 (Kesim 2023; biochemical definition) [11], and 47/51 (Gandhi 2024) [10]. The pooled clinical success was 97.6% (95% CI 83.4–99.7; I2≈64%), and the 95% prediction interval (≈53.6–99.9%) indicates that, while most settings are likely to achieve very high clinical response, variability in study definitions and patient mix can lead to wider uncertainty. Using crude totals, the overall proportion was 219/223 (98.2%).

#### Safety and adverse events

**Fig. 7:**
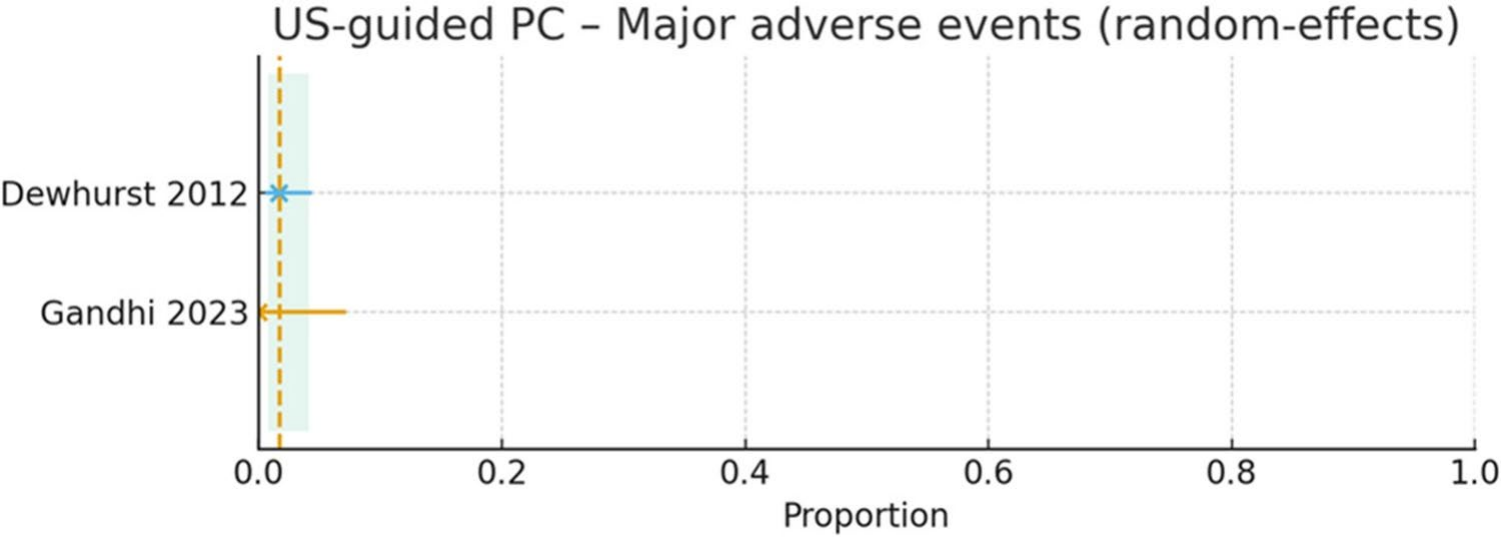
Study-level major adverse events after ultrasound-guided percutaneous cholecystostomy (US-guided PC), pooled with a random-effects model. Points show each study’s event rate with 95% CI (Dewhurst 2012; Gandhi 2023). The vertical dashed line marks the pooled proportion, and the shaded band its 95% CI. Major AEs were rare (≈0–2%) across cohorts; interpretation is limited by only two studies and varying AE definitions

Major adverse events (AEs) were reported by two cohorts (*n* = 293): 0/51 (Gandhi 2024) [10] and 4/242 (1.7%) in Dewhurst 2012 [9] (events included procedure‑related hemorrhage requiring transfusion, sepsis, biloma, and one procedure‑related death) (Fig. 7). The pooled major AE rate was 1.7% (95% CI 0.7–4.1; I2 = 0%). Minor/specific AEs included bile leak (1/51, Gandhi 2024) [10] and catheter dislodgement (3/27) and a post‑removal hepatic bleed (1/27) in Bakkaloglu 2006 [8]. Thirty‑day mortality was available from Dewhurst 2012 only [9]: 15/242 (6.2%), with one procedure‑related death and the remainder attributed to comorbidity.

#### Resource use and downstream management

Interval cholecystectomy after US‑PC was frequent where reported: 44/51 (86%) in Gandhi 2024 [10] and 68/145 (47%) in Kesim 2023 [11], while Bakkaloglu 2006 reported 5/27 (19%) undergoing delayed cholecystectomy [8]. Kesim 2023 explicitly noted that 51.7% of patients were managed definitively with US‑PC without additional biliary interventions [11].

### Heterogeneity, small‑study effects, and sensitivity

Between‑study statistical heterogeneity was negligible for technical success and major AEs (I2≈0%), but moderate to high for clinical success (I2≈64%), largely explained by differences in outcome definitions. Given the limited number of studies per outcome (*k* < 10), formal small‑study effect testing and funnel plots were not performed.

### Certainty of evidence

We assessed the certainty of evidence using GRADE for the critical outcomes. Given that all included studies were non-randomized observational cohorts, certainty started at low and was rated down where applicable for risk of bias, inconsistency, indirectness, and imprecision. The Summary of Findings table reports pooled estimates and judgments.

**Technical success –** Low certainty. Observational design with serious risk of bias (ROBINS‑I), but consistent results across studies (I2≈0%). Imprecision was limited due to narrow confidence intervals around very high proportions. No major indirectness.

**Clinical success –** Very low certainty. Serious risk of bias and serious inconsistency (I2≈64%) primarily due to heterogeneous definitions (symptom-based vs biochemical thresholds at ~ 72 h). Some indirectness in outcome definition across studies.

**Major adverse events –** Low certainty. Serious risk of bias but low inconsistency (I2≈0%). Imprecision remains possible given the small number of events and only two contributing studies.

**30-day mortality –** Very low certainty. Evidence comes from a single cohort; precision and consistency cannot be adequately assessed; risk of bias serious. This outcome was not pooled.

### Exploratory comparison: US-guided vs CT-guided PC

**Fig. 8:**
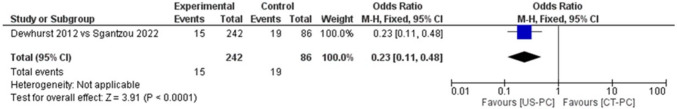
Forest plot comparing 30-day mortality after percutaneous cholecystostomy performed under ultrasound guidance (US-PC) versus CT guidance (CT-PC). Data combine two single-centre cohorts (Dewhurst 2012; Sgantzou 2022) as a pseudo-trial: US-PC 15/242 deaths (6.2%) vs CT-PC 19/86 deaths (22.1%). The pooled fixed-effect odds ratio is 0.23 (95% CI 0.11–0.48), favouring US-PC. Interpretation should be cautious: estimates are crude and unadjusted, and the cohorts differ in case-mix/indication, yielding very low-certainty evidence

We compared single-center cohorts of US-guided PC (Dewhurst 2012) [9] and CT-guided PC (Sgantzou 2022) [7] (Table 2). Technical success was near-universal in both modalities (≈100%). US-PC reported a 30-day mortality of 6.2%, whereas CT-PC reported 22.1%. Major adverse events were rare in US-PC (1.7%) but more diverse in CT-PC (catheter dislodgement 10.5%, bleeding 8.1%, abscess 5.8%, pneumothorax 2.3%, peritonitis 1.2%, bowel perforation 1.2%) [7]. The crude odds ratio for 30-day mortality favored US-PC (OR 0.23, 95% CI 0.11–0.48), but these results are confounded by indication and non-comparable case-mix [7, 9] (Fig. 8). Certainty of evidence is very low.

**Table 2:** Outcomes of ultrasound-guided vs CT-guided percutaneous cholecystostomy from two single-centre cohorts

Modality	Study	Technical success	Major AES	30-day mortality
US-guided PC	Dewhurst 2012 [9]	242/243 (99.6%)	4/242 (1.7%)	15/242 (6.2%)
CT-guided PC	Sgantzou 2022 [7]	86/86 (100%)	9 dislodgements (10.5%), 7 bleed (8.1%), 5 abscess (5.8%), 2 pneumothorax (2.3%), 1 peritonitis (1.2%), 1 perforation (1.2%)	19/86 (22.1%)

Technical success was near-universal in both series; 30-day mortality was 6.2% with US-guided PC (Dewhurst 2012) versus 22.1% with CT-guided PC (Sgantzou 2022). Major adverse events were 1.7% overall in the US cohort, while the CT cohort reported category-specific events (catheter dislodgement 10.5%, bleeding 8.1%, abscess 5.8%, pneumothorax 2.3%, peritonitis 1.2%, bowel perforation 1.2%). PC = percutaneous cholecystostomy; clinical success not reported in these two cohorts. Estimates are crude and unadjusted; case-mix differences may confound comparisons

## Discussion

This review shows that US‑PC achieves very high technical success (~ 99%) with low rates of major adverse events (~ 1–2%) in adults with acute cholecystitis who are poor candidates for urgent cholecystectomy. Clinical response was also high but varied across studies due to heterogeneous definitions (symptom-based vs. biochemical surrogates). The consistency of technical performance across centers—including an ICU bedside cohort—supports the reliability of US guidance for gallbladder access.

In real-world high‑risk populations, US‑PC offers rapid source control with minimal invasiveness [14, 15]. A transhepatic route predominated and may confer catheter stability with low bile‑leak rates, which is relevant when procedures are performed at the bedside. Where surgical risk remains prohibitive, many patients can be managed definitively with US‑PC; when risk improves, US‑PC serves as a bridge to interval cholecystectomy. Our pooled data suggest that, for appropriately selected patients and experienced teams, US‑PC is a dependable component of the care pathway.

Direct, US‑specific head‑to‑head evidence versus CT‑guided PC [7] or EUS‑guided gallbladder drainage is limited [16, 17]. EUS‑GBD has shown favorable outcomes in some comparative literature, but device availability and operator expertise vary widely, particularly outside tertiary endoscopy units [16, 18, 19]. CT‑guided PC remains an option when sonographic windows are poor; however, in many acute care settings, US is faster, portable, and radiation‑free [7]. Future trials should compare these modalities with standardized endpoint definitions and cost‑effectiveness assessments.

Given the high technical success and acceptable safety profile, US‑PC should remain readily available for high‑risk patients, ideally within a protocolized pathway that defines indications (e.g., Tokyo II–III or sepsis), antibiotic stewardship, early reassessment for surgery, and catheter management (size, route, dwell time, and removal criteria) [4, 20, 21]. Training and credentialing for ICU‑bedside performance could further improve access and timeliness.

## Limitations of the study

This synthesis has several limitations. First, the evidence is based on non-randomized single‑arm cohorts; ROBINS‑I judgments were generally at serious risk of bias, mainly due to confounding and selection processes. Clinical success was heterogeneously defined (symptom‑based vs biochemical thresholds), introducing inconsistency and indirectness. Moreover, outcome reporting was incomplete or heterogeneous across studies (e.g., denominators differed for technical success vs complications; specific adverse events were variably defined). Mortality at 30 days was available from a single cohort and was not meta‑analyzed. Furthermore, all studies were single‑center and mostly transhepatic, limiting generalizability to centers favoring transperitoneal access or with different operator expertise. Finally, small numbers of studies per outcome precluded robust assessment of small‑study effects and publication bias.

## Unresolved issues and areas of uncertainty in ultrasound-guided percutaneous cholecystostomy

Despite the high technical success and favorable safety profile of US-PC, several clinically relevant issues remain unresolved. First, the definition of clinical success is highly heterogeneous across studies, ranging from symptom resolution to biochemical response within variable timeframes. This lack of standardization limits between-study comparability and contributes to the observed heterogeneity in pooled estimates. A consensus definition incorporating both clinical and laboratory parameters, as well as a standardized assessment window, is still lacking. Moreover, optimal patient selection remains unclear. Although US-PC is commonly offered to patients with Tokyo grade II–III acute cholecystitis, Tokyo severity alone does not fully capture surgical risk, frailty, or reversibility of organ dysfunction [1, 2, 22]. Prospective studies integrating validated frailty scores and peri-procedural risk stratification tools are needed to refine indications. Third, technical aspects of the procedure require further clarification, including the choice between transhepatic and transperitoneal access, optimal catheter caliber, and standardized catheter management protocols. While the transhepatic route is often favored for stability and reduced bile leak, high-quality comparative data are sparse. Furthermore, the role of US-PC as definitive therapy versus a bridge to delayed cholecystectomy remains debated [21]. Reported rates of interval cholecystectomy vary widely, reflecting heterogeneity in patient fitness, institutional pathways, and follow-up strategies [23]. Identifying predictors of patients who may safely avoid surgery after drainage remains an unmet need. Finally, comparative effectiveness data between US-PC, CT-guided PC, and endoscopic ultrasound-guided gallbladder drainage are limited, and existing studies are often confounded by indication [18]. Well-designed prospective and randomized studies with standardized outcomes are required to address these gaps.

## Conclusions

Across contemporary cohorts, US-PC achieves very high technical success (~ 99%) and low major complication rates (~ 1–2%) in high-risk adults with acute cholecystitis [8–11]. Clinical success is generally high, but estimates vary because definitions (symptom-based vs biochemical) are heterogeneous. When contrasted descriptively with a single CT-guided cohort, 30-day mortality appeared lower with US-PC [OR: 0.23 (95% CI 0.11–0.48)], although this crude, indirect comparison is confounded by indication and non-comparable case-mix, and therefore should not be interpreted as proof of superiority [7, 9]. From a practical standpoint, US-PC offers bedside feasibility, avoids radiation and iodinated contrast, and supports rapid source control as either a bridge to interval cholecystectomy or, in selected patients, definitive therapy. The predominance of the transhepatic approach in included studies suggests good catheter stability with low bile-leak rates, including in ICU settings [10]. Future research should prioritize prospective, comparative studies (US-PC vs CT-PC and vs EUS-GBD), standardized endpoint definitions (especially for “clinical success” and major AEs), and adjusted analyses that account for frailty, severity (Tokyo grade), and procedural factors (access route, catheter size, bedside vs suite).

## Supplementary Information

Below is the link to the electronic supplementary material.

Supplementary file1 (DOCX 270 KB)

Supplementary file2 (DOCX 28 KB)

## Data Availability

Data are available upon request from authors.
