# Supplementary material for: Ultrasound-guided percutaneous cholecystostomy for acute cholecystitis: a systematic review and meta-analysis
Source: J Ultrasound. 2026 Feb 10;29(1):25–34. doi: 10.1007/s40477-026-01119-x (PMC13000085; doi:10.1007/s40477-026-01119-x)
Supplement: Supplementary file 2 — Supplementary file2 (DOCX 28 KB) [file 40477_2026_1119_MOESM2_ESM.docx]

# Search strategy

Databases and time frame. We searched MEDLINE/PubMed, EMBASE (Ovid), Web of Science Core Collection, and the Cochrane Library from inception to 26 Sep 2025, with no study-design filters at the search stage. Searches were limited to human studies; no language limits were applied in the search string, but we prespecified inclusion of articles in English or Italian (or with adequate English abstracts).

Concepts (PICO).

Population: adults with acute cholecystitis or gallbladder decompression candidates.

Intervention: ultrasound-guided percutaneous cholecystostomy (US-PC).

Comparator(s): CT-guided percutaneous cholecystostomy (CT-PC); any other modality reported in mixed cohorts was recorded.

Outcomes: technical success, clinical success, major adverse events, 30-day mortality (or nearest time-point).

Core search concepts and synonyms.

Procedure: cholecystostomy; percutaneous cholecystostomy; gallbladder drainage; gallbladder decompression.

Modality terms: ultrasound-guided; US-guided; sonography; ultrasonography; CT-guided; computed tomography.

Condition/context: acute cholecystitis; Tokyo Guidelines; high-risk surgical.

Example Boolean strategies (adapted per database syntax).

PubMed (MEDLINE)

(("Cholecystostomy"[Mesh] OR cholecystostomy[tiab] OR "percutaneous cholecystostomy"[tiab] OR "gallbladder drainage"[tiab] OR "gallbladder decompression"[tiab]) AND ((Ultrasonography[Mesh] OR ultrasound[tiab] OR ultrasonograph*[tiab] OR sonograph*[tiab] OR "US-guided"[tiab]) OR ("Tomography, X-Ray Computed"[Mesh] OR "computed tomography"[tiab] OR CT[tiab] OR "CT-guided"[tiab])) ) AND (humans[MeSH Terms])

EMBASE (Ovid)

(exp cholecystostomy/ OR cholecystostomy.ti,ab. OR percutaneous cholecystostomy.ti,ab. OR gallbladder drainage.ti,ab. OR gallbladder decompression.ti,ab.) AND ((exp ultrasonography/ OR ultrasound.ti,ab. OR ultrasonograph*.ti,ab. OR sonograph*.ti,ab. OR US-guided.ti,ab.) OR (exp computed tomography/ OR computed tomography.ti,ab. OR CT.ti,ab. OR CT-guided.ti,ab.)) AND [humans]/lim

Web of Science (Topic search)

TS=(cholecystostomy OR "percutaneous cholecystostomy" OR "gallbladder drainage" OR "gallbladder decompression") AND TS=(ultrasound OR ultrasonograph* OR sonograph* OR "US-guided" OR "computed tomography" OR CT OR "CT-guided")

Cochrane Library (Trials/CENTRAL)

(cholecystostomy OR "percutaneous cholecystostomy") AND (ultrasound OR ultrasonography OR sonography OR "US-guided" OR "computed tomography" OR CT OR "CT-guided")

Grey literature and supplementary searches. We screened reference lists of all included articles and relevant reviews; searched conference abstracts (e.g., DDW, ECR/WCR) within the time frame; and performed forward citation tracking in Google Scholar for key seed articles (Dewhurst 2012; Sgantzou 2022). Where necessary, we contacted corresponding authors for missing outcome data.

De-duplication and screening workflow. Records were exported to a citation manager and de-duplicated. Two reviewers independently screened titles/abstracts, then full texts, against prespecified eligibility criteria. Disagreements were resolved by consensus or adjudication by a third reviewer. We documented the process in a PRISMA flow diagram (identification, screening, eligibility, inclusion).

Protocol and registration. Methods followed PRISMA 2020 guidance.
